# Intimate Partner Homicide and Homicide–Suicide: Forensic Profiles and Clinical Correlates

**DOI:** 10.1192/j.eurpsy.2026.11281

**Published:** 2026-07-31

**Authors:** L. Mužinić Marinić, I. Marinić

**Affiliations:** Department of psychiatry, University hospital Dubrava, Zagreb, Croatia

## Abstract

**Introduction:**

Intimate partner homicide (IPH) and attempted homicide are among the most tragic outcomes of domestic violence, constituting a persistent social, forensic, and public health challenge. Despite global declines in homicide rates, the incidence of IPH has remained stable. Early recognition of risk factors and interdisciplinary prevention strategies are essential to mitigate fatal outcomes.

**Objectives:**

This study reviews current scientific and professional knowledge on perpetrators of IPH, with emphasis on forensic-psychiatric characteristics, socio-demographic profiles, prior violence and criminality, personality traits, substance use, depression, psychotic disorders, and the role of jealousy. Special attention is given to the association between depression, suicidal ideation, and the occurrence of homicide-suicide.

**Methods:**

A narrative literature review was conducted using PubMed for studies published between 2000 and 2024. Studies relevant to perpetrator characteristics of homicide and homicide-suicide delicts as well as risk factors were systematically examined.

**Results:**

Across studies, perpetrators are most often men of working age, with frequent histories of prior violence, substance misuse, or psychiatric difficulties. Personality traits such as borderline, narcissistic, and antisocial features are commonly observed, while severe mental illness is less frequent. Situational triggers—including separation, jealousy, and access to firearms—are repeatedly highlighted as critical risk factors.

**Conclusions:**

The forensic profile of IPH perpetrators reflects the interaction of individual vulnerabilities (personality traits, psychiatric disorders, substance use), relational dynamics (jealousy, prior abuse, separation), and situational triggers (alcohol intoxication, access to firearms). Effective prevention requires interdisciplinary collaboration among mental health, social services, police, and judicial systems, as well as public education to promote early recognition and protective interventions for victims. Timely therapeutic, social, and legal responses remain crucial to reducing the likelihood of the most severe forms of intimate partner violence.

**Disclosure of Interest:**

None Declared

